# Long-term outcomes and re-intervention rates in women undergoing mri-guided focused ultrasound (mrgfus) for uterine fibroids: a 7-year follow-up study

**DOI:** 10.1007/s10815-025-03405-9

**Published:** 2025-02-03

**Authors:** Yael Inbar, Jaron Rabinovici, Rachael Sverdlove, Tomer Ziv-Baran, Ronit Machtinger

**Affiliations:** 1 Department of Diagnostic Imaging, Sheba Medical Center, Ramat-Gan, Israel; 2 Department of Obstetrics and Gynecology, Sheba Medical Center, Tel-Hashomer, 52621 Israel, Ramat-Gan, Israel; 3 Faculty of Medical & Health Sciences, Tel-Aviv University, Tel-Aviv, Israel; 4 School of Public Health, Faculty of Medical & Health Sciences, Tel-Aviv University, Tel-Aviv, Israel

**Keywords:** Hysterectomy, Laparoscopy, MRgFUS, Myomectomy, Symptomatic uterine fibroids, Uterine artery embolization, Uterine leiomyoma

## Abstract

**Purpose:**

To assess the long-term outcomes of MR-guided focused ultrasound (MRgFUS) for treating uterine fibroids, focusing on re-intervention rates, pregnancy outcomes, and the onset of menopause over a 7-year follow-up period.

**Materials and Methods:**

We conducted a historical cohort study of 99 women with symptomatic uterine fibroids who underwent MRgFUS between 2013 and 2020 at a single tertiary medical center. Data collection included patient demographics, treatment details, and follow-up interviews. Re-intervention rates were evaluated using Kaplan–Meier curves and Cox regression analysis to identify predictors of further treatments, with a specific focus on age-related differences.

**Results:**

Over a median follow-up of 6.1 years, 33.1% of women required re-intervention for persistent fibroid symptoms. The median patient's age was 43 years old. Women aged ≤ 43 years had significantly higher re-intervention rates than those aged 44 + years (47.5% vs. 16.7%, p = 0.005). Multivariable Cox regression identified age as the sole significant predictor of re-intervention (HR_44+vs. <43_ 0.303 95% CI 0.128–0.714, p = 0.006). Sixteen women conceived after MRgFUS, resulting in 21 pregnancies, with 72.2% live births and a spontaneous miscarriage rate of 22.2%. The mean age of menopause was 51.4 years, similar to global averages.

**Conclusions:**

MRgFUS is a practical, noninvasive option for treating symptomatic uterine fibroids. Older women show lower re-intervention rates. Pregnancies post-MRgFUS are possible, and the procedure does not appear to affect the onset of menopause. Age remains a crucial predictor for further re-intervention.

## Introduction

Uterine fibroids are the most common, benign pelvic tumors in women, affecting up to 70% of reproductive-age women [1–3]. While many uterine fibroids can be clinically silent, approximately 30% of women require intervention because of significant symptoms, such as abnormal uterine bleeding and heavy menstrual bleeding, urinary symptoms, gastrointestinal discomfort, back pain, bulky symptoms, or infertility [4].

Treatments vary from medical therapies to surgical options like myomectomy or hysterectomy. More conservative approaches, such as uterine artery embolization (UAE) or thermo-ablative techniques like MRI-guided focused ultrasound (MRgFUS), are also available [5]. MRgFUS induces localized coagulative necrosis, identified with the non-perfused volume (NPV) (tissue without blood flow evaluated at the end of the treatment) [4, 6], which is correlated with fibroid shrinkage potential, making it a noninvasive and convenient option. However, it may lead to a need for further intervention [7–10]. Most follow-up studies last up to 5 years, with limited data on fertility outcomes and menopausal transition after the procedure. This analysis aimed to fill these gaps by assessing the annual re-intervention rates over 7 years in women who underwent MRgFUS. We investigated how age affected the likelihood of requiring additional procedures and evaluated pregnancy outcomes and the onset of menopause during the follow-up period.

## Materials and methods

According to our department's policy, only premenopausal women with uterine fibroids were eligible for MRgFUS treatment. We excluded from the analysis women who underwent MRgFUS due to adenomyosis. Until 2015, MRgFUS was recommended only for women who did not desire further fertility. Thus, women were advised not to conceive and to use reliable contraception. In 2015, the FDA approved a change in the labeling of MRgFUS, considering the procedure also in women with symptomatic uterine fibroids who desire to retain fertility and spare their uterus.

We reviewed the electronic charts of all women who underwent the procedure for symptomatic uterine fibroids at a single tertiary, university-affiliated Medical Center between 2013 and 2020.

The local institutional ethics committee approved the study (8769-21SMC). All participants gave their consent for follow-up phone interviews.

Data collected included patients' age at MRgFUS procedure, treatment date, gravida, parity, previous laparotomies, uterine fibroid symptom-quality of life-symptom severity score (UFS-SSS-QOL) before the procedure [11], fibroid-associated symptoms, comorbidities, desire for future fertility, number of MRgFUS treatments (1 or 2), fibroids' signal intensity on T2 weighted imaging, NPV of the fibroids at the end of the procedure, situations of which the patients asked to stop the treatment due to intractable pain, cases warming of the subcutaneous tissue at the end of the treatment and date of the last follow-up or the operation. Phone interviews were performed with all patients (R.M and Y.R), asking whether they underwent re-interventions (i.e., uterine artery embolization, hysteroscopic myomectomy, myomectomy by laparoscopy or laparotomy, or hysterectomy by laparoscopy, laparotomy or vaginally) for the persistence or recurrence of fibroid-associated symptoms, whether they tried to conceive following the procedure or entered menopause. In the case of menopause, women were asked about the age of their last menstrual period. Patients' medical records verified self-reported re-interventions. Women who did not undergo surgical intervention within seven years of follow-up were censored.

### Statistical analysis

Categorical variables were summarized as frequency and percentage, while continuous variables were reported as mean (+ SD). Patients' baseline characteristics and treatment data were compared using the Mann–Whitney (continuous and ordinal variables) or the Chi-square test / Fisher's exact test (categorical variables).

Follow-up was up to 7 years after the MRgFUS treatment, as the number of women with a complete follow-up of > 7 years was too small for the statistical analyses (19%). The reverse censoring method was used to evaluate the length of follow-up. Kaplan–Meier curves were applied to describe the need for surgical intervention during the follow-up period. We used a binary cut-off of 44 years old to assess the effect of the patient's age on the rate of surgical interventions following MRgFUS treatment, as this was the median population age. Moreover, the mean age of menopause is expected to be 51 years old, and we assumed that fibroids' associated symptoms would be less relevant post-menopause. A log-rank test was used to compare the need for surgical intervention between the two age groups.

Multivariable Cox regression was used to identify independent predictors for surgical invention during the follow-up period. Age (below or above median), gravida, parity, fibroid volume, signal intensity on T2 weighted imaging (hypo-intense vs. other), non-perfused volume at the end of the treatment, and number of MRgFUS treatments were considered as potential predictors. The forward stepwise method was used for variable selection (p < 0.1 at Wald test was used as criteria for inclusion).

All statistical tests were two-sided. P-value < 0.05 was considered statistically significant. All statistical analyses were performed using the SPSS software (IBM SPSS Statistics for Windows, version 28, Armonk, NY, USA).

## Results

From 2013 to 2020, 100 women with a history of uterine fibroids underwent MRgFUS in our hospital. Patients were followed up to 3 times after the procedure; the last tracking was performed in August 2024. One was lost for follow-up immediately following the treatment, and 99 were included in this analysis. The mean patients' age + SD was 42.3 + 5.7 years old. The median patient's age was 43 years old. The median follow-up time was 6.1 years. Seventeen women (17.2%) underwent at least one previous laparotomy before MRgFUS treatment. Patients > 44 years had significantly more urinary symptoms compared with the younger patients (< 43 years old) (51.1% vs. 23.1%, p = 0.004). NPV% > 70% was achieved by 70% of the participants. There were no differences regarding the number of fibroids, fibroids' volume, hypo-intensity in T2 weighted imaging, or NPV% at the end of the treatment between the groups. Patients' demographics are shown in Table 1.

**Table 1:** Patients' demographics

	**All**	** ≤ 43 years**	** > 44 years**	**p**
	**N = 99**	**N = 52**	**N = 47**	
**Age (years)**: Mean (SD) **UFS-QOL-SSS**: Mean (SD)	43.0 (5.6) 25.8 (6.3)	38 (4.2) 25.1 (6.4)	47.0 (2.2) 26.5 (6.2)	0.346
**Gravida**: Median (IQR)	2.0 (0.0–3.0)	1.0 (0.0–2.0)	2.0 (2.0–3.0)	< 0.001
**Parity**: Median (IQR)	2.0 (0.0–3.0)	0.0 (0.0–2.0)	2.0 (1.0–3.0)	< 0.001
**Previous laparotomy**, n (%) **Urinary symptoms,** n (%) **Bleeding symptoms,** n (%) **Pain,** n (%) **Desire to maintain fertility,** n (%) **Patients with associated Comorbidities,** n (%) **Number of fibroids** Mean (SD) **Hypo intense fibroid in T2,** n (%) **Fibroids' volume (cm**^**3**^**)** Mean (SD) **A single MRgFUS treatment,** n (%) **Non perfused volume (NPV)%** at the end of the treatment Mean (SD)	17 (17.2%) 36 (36.4%) 75 (75.8%) 27 (27.3%) 35 (35.4%) 23 (23.2%) 1.6 (1.6) 65 (65.7%) 166.5 (112.4) 90 (90.9%) 76 (20.1)	6 (11.5%) 12 (33.3%) 41 (54.7%) 13 (25.0%) 35 (67.3%) 12 (23.0%) 1.4 (0.8) 35 (67.3%) 166.1 (93) 44 (84.6%) 75.4 (22.8%)	11 (23.4%) 24 (66.7%) 34 (45.3%) 14 (29.8%) 0 (0%) 11 (23.4%) 1.7 (2.2) 30 (63.8%) 167.0 (131.2) 46 (97.9%) 76.6 (16.8%)	0.118 0.004 0.451 0.593 < 0.001 0.267 0.720 0.716 0.594 0.033 0.779
**Stopped the treatment due to intractable pain,** n (%) **Warming of subcutaneous tissue,** n (%)	8 (8.1%) 10 (10.1%)	6 (11.5%) 5 (9.6%)	2 (4.3%) 5 (10.6%)	0.274 1

*IQR* inter quartile range, *SD* standard deviation, *UFS-QOL-SSS* uterine fibroid symptom-quality of life-symptom severity score

Thirty-three percents of the patients who underwent MRgFUS for symptomatic uterine fibroids underwent re-interventions for the persistence of uterine fibroids-associated symptoms during a follow-up of up to 7 years. Women who were 44 years old or older had statistically significantly lower chances of undergoing re-intervention during the follow-up period than those who were ≤ 43 years old at the time of the MRgFUS treatment (16.7% vs. 47.5%, p = 0.005).

Of those women who underwent re-intervention, nineteen women (19.2%) underwent laparoscopic, open myomectomy, or hysterectomy during this period, seven (7.1%) underwent hysteroscopic myomectomy, and one woman (1%) underwent uterine artery embolization.

**Fig. 1:**
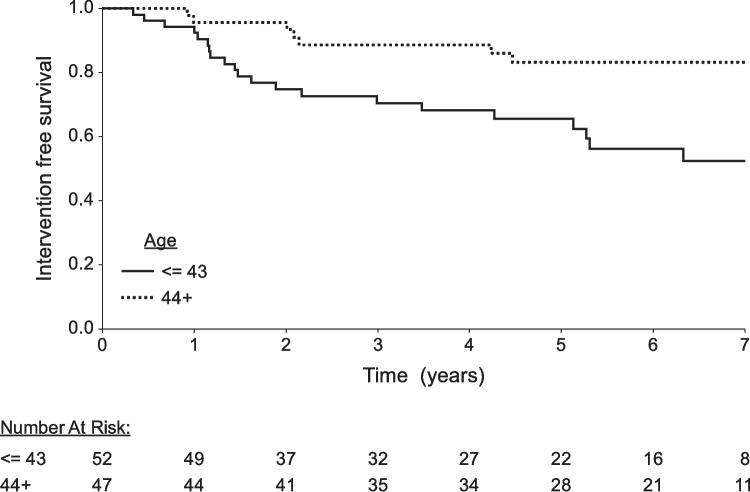
Kaplan–Meier curves describing the need for surgical interventions during the follow-up period

For patients ≤ 43 years, at year 1, the rate of re-interventions was 7.7%, which increased to 47.5% by year 7 (Fig. 1). For patients > 44 years, at year 1, the rate of surgical interventions was 4.3%, which increased to 16.1% by year 7. Like the younger age group, there was a steady increase over time. Using Cox regression, only age was identified as a predictor for any intervention (HR > 44 vs. < 43; 0.303 95% CI 0.128–0.714, p = 0.006). Other variables did not reach statistical significance.

During the follow-up, 20 women out of 52 women < 43 years old (38.4%) reported that they tried to get pregnant. Thirteen of them (65%) further conceived after the procedure and achieved eighteen pregnancies. One patient conceived four times, two patients conceived twice during the follow-up time, and ten conceived once. Four pregnancies (22.2%) ended due to first trimester spontaneous miscarriages.

Thirteen pregnancies (72.2%) ended in deliveries. Eight pregnancies ended in spontaneous deliveries and five in cesarean sections. There was one case of intrauterine growth retardation and one case of postpartum hemorrhage. One case was lost to follow-up after 21 weeks of gestation.

Another three patients had unintended pregnancies. One of them, > 44 years old, had a molar pregnancy. The other two, from the younger group, underwent artificial abortions.

Eighty women who did not undergo hysterectomy or open/laparoscopic myomectomy were asked about menopause. Three refused to answer. Twenty-three (29.8%) reported that they were menopausal. The mean age of menopause + SD was 51.4 + 2.7 years.

## Discussion

Over seven years of follow-up, the cumulative rates of interventions for persistent uterine fibroid symptoms were 33.1%. Multivariable Cox regression to identify independent predictors for re-invention during the follow-up period showed that only age influenced the need for further treatments. Women who were 44 years old or older had significantly lower chances of undergoing re-intervention compared with those who were ≤ 43 years old at the time of the MRgFUS. Of 20 women who tried to conceive, 13 became pregnant (18 pregnancies). Another three women had unintended pregnancies. The mean age of menopause (self-reported) was 51 years old, similar to the global population.

A recent meta-analysis by Xu et al. summarized the re-intervention rates for one year, two years, three years, and five years for women who underwent MRgFUS for uterine fibroids. Based on several studies, intervention rates were 12% (n = 442), 14% (n = 91), 22% (n = 329), and 49% (n = 336) after one year, two years, three years, and five years, respectively [12]. While the intervention rates in the meta-analysis after two years and three years were similar to our study (14.0% and 22.0% vs. 15.4% and 20.9%, respectively), the need for further interventions in our cohort after one year and five years (6.1% and 26.1%, respectively) were lower than reported by Xu et al., (12% and 49%). Possible reasons for the different results include the patients' age distribution or the genetic background of the different patient populations that might have affected treatment outcomes. Another systematic review and a meta-analysis by Duo et al. reported re-intervention rates of 12% after 1 year, 12% after 2 years 29% after 3 years and 53%. The rates are higher than those reported in our cohort, as Dou et al. analysis also includes studies performed before 2010 [13]. During the older period, MRgFUS was only allowed to ablate 33%−50% of the fibroid volume, less than 100mm^3^−150 mm^3^ of a single fibroid, less than 120–180 min of the operation time, thus limiting the possibility of higher NPV at the end of the treatment, leading to a higher re-intervention rate.

Gorny et al. followed 138 women for 1–7.2 years. The mean length of follow-up was 2.8 years. Re-intervention rates after 3 and 4 years from the MRgFUS treatments were very similar to our study – 19% and 23%, respectively. Older women, as women who had fibroids that were not heterogeneous or bright fibroids on T2-weighted MR imaging, required less re-intervention [14]. Although in our analysis only age was identified as a predictor for re-intervention, when we added a sub-analysis looking only at the rates of major intervention (only cases of hysterectomy and myomectomy), both age (HR > 44 vs. < 43; 0.289 95% CI 0.105–0.799, p = 0.017) and dark (hypo-intensive) fibroids on T2-weighted imaging (hypo-intensive vs. heterogeneous or bright 0.368 95% CI 0.152–0.891, p = 0.027) were significant. Li et al. also found a significant correlation between patients' age and signal intensity on the uterine fibroids' T2-weighted image (T2WI) [15].

The younger age group seeking more interventions has several possible explanations. Women above 44 had more children on average than the younger cohort (p < 0.001). A potential desire for future children could explain why younger women sought further interventions after MRgFUS treatment. The presence of fibroids can affect conception and pregnancy, which might have made the younger cohort more willing to undergo interventions.

Another possible explanation could be that the older women had a higher rate of previous surgeries before the MRgFUS treatment (p < 0.001). Laparotomy carries several risks and side effects, such as bleeding, pain, and adhesion formation that affect the technical possibilities of the procedure. As women > 44 yrs. old had undergone more interventions compared with those < 43 yrs. old, they might have been more reluctant to undergo another surgery after MRgFUS. Another possible reason is that many older women may have entered menopause. Thus, the fibroids became asymptomatic.

Thirteen women from our cohort underwent 18 pregnancies. Around 72% ended in live births. The rate of spontaneous miscarriages was 22.2%. A study by Rabinovici et al., published in 2010, reported the world experience of pregnancies after MRgFUS treatment (reports from 13 sites in seven countries). Fifty-one women achieved 54 pregnancies. Forty-one percent (22 of 54) of pregnancies resulted in deliveries, and 20% (11 of 54) were ongoing beyond 20 weeks. The miscarriage rate was 26% (14 of 54) [16]. Fourteen studies reported 124 pregnancies after MRgFUS. A recent systemic review by Anneveldt et al. [17] reported 73% of live birth, similar to our study and to Rabinovici study. Interestingly, the cesarean section rate in our study was 36% much lower than previously reported. According to this data, post-procedure, pregnancy is possible, but to date, there is a lack of evidence that this procedure can serve as a fertility-enhancing treatment. As data regarding long-term obstetrical complications, antepartum, and postpartum complications are still limited, we recommend that if the patient is of reproductive age and does not want to get pregnant, reliable contraception should be used.

The mean age of menopause reported in our cohort was 51.4 + 2.7 years, which seems similar to the global mean age of menopause. To the best of our knowledge, no previous studies evaluated the effect of MRgFUS on the age of menopause or ovarian reserve. However, several reports of permanent loss of ovarian function after uterine fibroid embolization, specifically in women > 45 years old, exist [18–20].

The main strength of our study is the ability to get a complete follow-up of most of the patient's treatment, with a minimal number of women lost for follow-up. In addition, since the procedure has been performed in our hospital since 2003, and all treatments were done by a single and very experienced radiologist (Y.I.), no learning curve was expected. Our study has some limitations. Firstly, a larger sample size than the 99 patients described here might be more representative of the entire population of women suffering from uterine fibroids. Second, since our study was conducted at a single tertiary center, our patient findings may need to be more generalizable to other populations. Third, the number of women who planned to conceive in our cohort and achieved pregnancy is too small, thus enabling us to give descriptive information only. It would be ideal to have a larger sample size and also to compare pregnancy rates and outcomes to control groups of women of the same age group without fibroids and those with untreated fibroids attempting to conceive. However, this is beyond the scope of the current study. In addition, there might be a recall bias regarding patients' age of menopause, as data was self-reported.

In conclusion, our research provides important insights into the long-term outcomes of MRgFUS treatment for uterine fibroids, specifically concerning re-intervention rates, pregnancy outcomes, and the menopausal transition. Our findings show that age is a significant predictor of the likelihood of requiring further surgical interventions, with women over 44 years showing a lower necessity for additional procedures compared to younger women. Nonetheless, MRgFUS remains a promising, noninvasive option for managing symptomatic uterine fibroids, particularly in older women. Further studies need to be done assessing pregnancy outcomes following MRgFUS among women desiring future fertility.
